# Supplementary material for: Content-rich biological network constructed by mining PubMed abstracts
Source: BMC Bioinformatics. 2004 Oct 8;5:147. doi: 10.1186/1471-2105-5-147 (PMC528731; doi:10.1186/1471-2105-5-147)
Supplement: Additional File 2 — The original results of the above study (non-essential files are deleted to keep the file size under the limit set by BMC bioinformatics). [file 1471-2105-5-147-S2.bz2 › chilibotAdditionalFile2/dip50/371ID10950949E6046/html/FGF2_FGFR1.html]

 


 **FGF2** and **FGFR1** 
  
Found 575 abstracts in PubMed, retrieved 50.  
 

 What does Google say? 
 PDF only 
| .edu only 

---

**Interactive relationship** (e.g. stimulation, inhibition, etc)

**Stimulatory relationship**- FGF 2  [ **FGF2** ]  stimulation induced translocation of the FGF type I receptor  **FGFR1**  into nuclei, suggesting that the growth stimulating effect of FGF 2  [ **FGF2** ]  was mediated through  **FGFR1** .  Ref: 12513125 In Vitro Cell Dev Biol Anim, 2002
- In situ hybridization analyses of TRalpha knockout mice, which have impaired ossification and skeletal mineralization, revealed reduced  **FGFR1**  mRNA expression in osteoblasts and osteocytes, whilst T3 failed to stimulate  **FGFR1**  mRNA or enhance  **FGF2**  activated MAPK signaling in TRalpha null osteoblasts.  Ref: 12805413 Mol Endocrinol< MedlineTA>Mol Endocrinol, 2002
- Analysis of tryptic phosphopeptide maps of FGFR 1  [ **FGFR1** ]  from cells stimulated with FGF 2  [ **FGF2** ]  alone and FGF 2  [ **FGF2** ]  together with heparin showed that FGF 2  [ **FGF2** ]  alone stimulated a several fold increase in tyrosine 463 in the juxtamembrane domain.  Ref: 12799194 Exp Cell Res, 2003
- These enhanced responses to bFGF  [ **FGF2** ]  appear to be due to increased expression of fibroblast growth factor receptor 1  [ **FGFR1** ] , at both the mRNA and protein levels, in the cyclin D1 overexpressing cells.  Ref: 12543798 Cancer Res, 2003
- Isolated CD34 FGFR  [ **FGFR1** ]  cells grow slowly in culture, are stimulated by fibroblast growth factor 2  [ **FGF2** ]  and vascular endothelial growth factor, and give rise to cells that express von Willebrand factor and VE cadherin.  Ref: 12411316 Blood, 2002
- Increased amounts of HSPG were present in the dermal nodules of scleromyxedema compared to adjacent normal dermis and these bound FGF 2  [ **FGF2** ] , immobilized the soluble receptor protein FGFR 1  [ **FGFR1** ]  and, therefore, formed a ternary complex composed of HSPG, FGF 2  [ **FGF2** ]  and FGFR 1  [ **FGFR1** ]  in vitro.  Ref: 12498227 Pathol Res Pract, 2002
- Hence, FGF 2  [ **FGF2** ]  attached to the heparan sulfate moiety expressed by the novel CD44 variant of RA synovium cells exhibits an augmented ability to stimulate FGFR 1  [ **FGFR1** ]  mediated activities.  Ref: 12697740 J Clin Invest, 2003
**Neutral relationship**- We find that SMC and BAEC express the same FGFR  [ **FGFR1** ]  isoforms and bind  **FGF2**  with equal affinity at the cell surface, yet FGF has a markedly higher proliferative effect on SMC than on BAEC.  Ref: 12659634 Biochem J< MedlineTA>Biochem J, 2003
- Our studies have shown that full length FGF receptor 1  **FGFR1**  accumulates within the nuclear interior in parallel with FGF 2  [ **FGF2** ] .  Ref: 12647309 J Cell Biochem, 2003
- Our data suggest that low oxygen upregulates  **FGFR1**  expression in embryonic aorta in vitro and renders it more responsive to  **FGF2** .  Ref: 12388106 Am J Physiol Cell Physiol, 2003
- However, Namalwa cells, immobilizing FGF 2  [ **FGF2** ]  via their cell surface CD44vRA, bound substantially more soluble FGF receptor 1 FGFR 1  [ **FGFR1** ]  than did Namalwa cells immobilizing the same amount of FGF 2  [ **FGF2** ]  via their cell surface CD44v3 v10.  Ref: 12697740 J Clin Invest, 2003
- The biological effects of FGF 2  [ **FGF2** ]  in the myocardium are mediated by the high affinity tyrosine kinase receptor FGFR 1  [ **FGFR1** ] , the major FGF receptor in the heart.  Ref: 12504809 Cardiovasc Res, 2003
- The biological activity of bFGF  [ **FGF2** ]  is mediated through interaction with its high affinity receptor, fibroblast growth factor receptor 1  [ **FGFR1** ]  FGFR 1  [ **FGFR1** ] .  Ref: 12651849 J Biol Chem< MedlineTA>J Biol Chem, 2003
- These cells were also primarily FGFR 1  [ **FGFR1** ] , with few EGFR, and most expanded and progressed along their expected lineages in bFGF  [ **FGF2** ]  containing medium but not in EGF containing medium.  Ref: 12514221 J Neurosci, 2003
- In vitro assays demonstrate that specific glycopolymers can potentiate FGF 2  [ **FGF2** ]  FGFR 1  [ **FGFR1** ]  binding interactions.  Ref: 12440867 Bioconjug Chem< MedlineTA>Bioconjug Chem, 2003
- The biological significance of VEGF, EGF, and bFGF  [ **FGF2** ]  is mediated by their receptors, which belong to the family of tyrosine kinase receptors Flt 1 VEGFR 1, KDR VEGFR 2, EGFR, FGFR 1  [ **FGFR1** ] , FGFR  [ **FGFR1** ]  2, FGFR  [ **FGFR1** ]  3, and FGFR  [ **FGFR1** ]  4.  Ref: 12500263 Microsc Res Tech, 2003
- Exogenous FGF 2  [ **FGF2** ]  decreased  **FGFR1**  protein.  Ref: 12746216 Am J Physiol Endocrinol Metab< MedlineTA>Am J Physiol Endocrinol Metab, 2003

**Non-interactive relationship** (e.g. studied together, co-existance, homology, etc.)

- The objective of this study was to elucidate the correlation between the expression of FGF 2  [ **FGF2** ] , VEGF, and their receptors FGF receptor 1 FGFR 1  [ **FGFR1** ]  and VEGF receptor 1 Flt 1 with microvessel density in synovial tissues of the TMJ.  Ref: 12651930 J Dent Res, 2003
- The possible roles of FGF 2  [ **FGF2** ]  and FGFR 1  [ **FGFR1** ]  in SC of the TMJ are discussed.  Ref: 12418570 Int J Oral Maxillofac Surg, 2002
- The aim of this study was to assess the subcellular localization of basic fibroblast growth factor  [ **FGF2** ]  bFGF  [ **FGF2** ]  and fibroblast growth factor receptor 1  [ **FGFR1** ]   **FGFR1**  in pituitary adenomas.  Ref: 12455885 Brain Tumor Pathol, 2002
- Mono and disaccharide containing glycopolymers were synthesized by two different free radical processes, and their ability to act as heparan sulfate glycomimetics in promoting the binding of Fibroblast Growth Factor 2  [ **FGF2** ]  FGF 2  [ **FGF2** ]  to its receptor FGFR 1  [ **FGFR1** ]  was evaluated using an in vitro cell based assay.  Ref: 12440867 Bioconjug Chem< MedlineTA>Bioconjug Chem, 2002
- Colocalisation of  **FGFR1**  with FGF 2  [ **FGF2** ]  could not be demonstrated in unstressed retina, but was demonstrable in stressed retina, in both the ONL and OPL.  Ref: 12708794 Growth Factors, 2002
- The immunohistochemistry for bFGF  [ **FGF2** ] ,  **FGFR1** , and MIB 1 was examined in paraffin embedded tissues.  Ref: 12455885 Brain Tumor Pathol, 2002
- We also measured components of the FGF 2  [ **FGF2** ]  ternary signaling complex, fibroblast growth factor receptor 1  [ **FGFR1** ]  FGFR 1  [ **FGFR1** ]  and syndecan 4.  Ref: 12832283 Am J Physiol Lung Cell Mol Physiol< MedlineTA>Am J Physiol Lung Cell Mol Physiol, 2002
- The expression of fibroblast growth factor 2  [ **FGF2** ]  FGF 2  [ **FGF2** ]  and fibroblast growth factor receptor 1  [ **FGFR1** ]  FGFR 1  [ **FGFR1** ]  in two cases of SC of the temporomandibular joint TMJ were immunohistochemically studied.  Ref: 12418570 Int J Oral Maxillofac Surg, 2002
- DNFR reduced glioma cell accumulation with apoptosis and this reduction was alleviated with exogenous epidermal growth factor EGF, which can activate Ras independent of FGFR  [ **FGFR1** ]  but not with bFGF  [ **FGF2** ] .  Ref: 12430714 J Neuropathol Exp Neurol, 2002
- Similarly, T3 enhanced  **FGF2**  activated autophosphorylation of  **FGFR1** , but did not modify  **FGF2**  induced phosphorylation of the docking protein FRS2.  Ref: 12805413 Mol Endocrinol< MedlineTA>Mol Endocrinol, 2002
- FGF 2  [ **FGF2** ]  alone stimulated maximal phosphorylation of Src homology domain 2 tyrosine phosphatase SHP 2 and the adaptor molecule Crk, in heparan sulfate deficient Chinese hamster ovary CHO 677 cells expressing FGFR 1  [ **FGFR1** ] .  Ref: 12799194 Exp Cell Res, 2003
- The mean expression of VEGF, bFGF  [ **FGF2** ] , FLK 1 and FLG  [ **FGFR1** ]  of axillary lymph node positive patients was higher than the negative ones both in young and postmenopausal women groups P <.  Ref: 12795839 Zhonghua Zhong Liu Za Zhi, 2003
- Thyroidal levels of fibroblast growth factor 2  [ **FGF2** ]  FGF 2  [ **FGF2** ]  and fibroblast growth factor receptor 1  [ **FGFR1** ]   **FGFR1**  are elevated in human thyroid hyperplasia.  Ref: 12746216 Am J Physiol Endocrinol Metab< MedlineTA>Am J Physiol Endocrinol Metab, 2003
- The FGF FGFR  [ **FGFR1** ]  stimulated 6xOSE2 promoter activity was also blocked by inhibiting PKC, as was the  **FGF2**  stimulation of the DNA binding activity of Runx2.  Ref: 12403780 J Biol Chem, 2003
- Using semiquantitative reverse transcription polymerase chain reaction, we examined the expression of insulin like growth factor 1 Igf 1, fibroblast growth factor 2  [ **FGF2** ]  Fgf 2  [ **FGF2** ] , and their receptors Igf 1r,  **Fgfr1** , Fgfr2, and Fgfr3 in the MCC of 28 day old rats at, and 14 days after placing intraoral appliances designed to produce a lateral functional shift of the mandible.  Ref: 12594422 Am J Orthod Dentofacial Orthop, 2003
- Increases in  **FGFR1**  or FGF 2  [ **FGF2** ]  and both may contribute to goitrogenesis.  Ref: 12746216 Am J Physiol Endocrinol Metab< MedlineTA>Am J Physiol Endocrinol Metab, 2003
- Neither cytoplasmic bFGF  [ **FGF2** ] , cytoplasmic  **FGFR1** , nor MIB 1 staining index showed any relationship with the recurrence of pituitary adenomas.  Ref: 12455885 Brain Tumor Pathol, 2002
- We have studied the mechanism whereby heparin directs FGF 2  [ **FGF2** ]  induced FGF receptor 1 FGFR 1  [ **FGFR1** ]  signal transduction.  Ref: 12799194 Exp Cell Res, 2003
- We assessed the role of fibroblast growth factors FGF in pulmonary vascular remodeling by examining the expression pattern of FGF 1, FGF 2  [ **FGF2** ] , and the FGF receptor FGFR 1  [ **FGFR1** ]  in peripheral area of lung tissues from patients with COPD FEV 1 <.  Ref: 12397010 Am J Respir Cell Mol Biol, 2002
- These changes are associated with specific alterations in the regulation of FGF 2  [ **FGF2** ] , FGFR 1  [ **FGFR1** ]  and syndecan 4 in the airway epithelial mesenchymal trophic unit, which may be associated with the developmental problems of the lung associated with exposure to ozone.  Ref: 12832283 Am J Physiol Lung Cell Mol Physiol< MedlineTA>Am J Physiol Lung Cell Mol Physiol, 2002
- Finally, isolated primary synovial fluid cells from RA patients expressing CD44vRA bound more soluble FGFR 1  [ **FGFR1** ]  to their cell surface associated FGF 2  [ **FGF2** ]  than did corresponding synovial cells expressing CD44v3 v10 or synovial cells from osteoarthritis patients.  Ref: 12697740 J Clin Invest, 2003
- The expression of fibroblast growth factor 2  [ **FGF2** ]  and fibroblast growth factor receptor 1  [ **FGFR1** ]  in chondrocytes in synovial chondromatosis of the temporomandibular joint.  Ref: 12418570 Int J Oral Maxillofac Surg, 2002
- **FGFR1**  expression and  **FGFR1**  FGF 2  [ **FGF2** ]  colocalisation in rat retina sites of FGF 2  [ **FGF2** ]  action on rat photoreceptors.  Ref: 12708794 Growth Factors, 2002
- METHODS The expression of VEGF, FLK 1, bFGF  [ **FGF2** ]  and FLG  [ **FGFR1** ]  in 40 young and 30 postmenopausal women with breast cancer was studied by immunohistochemical method SABC, with its relation with axillary lymph node metastasis and the clinical and pathologic characteristics.  Ref: 12795839 Zhonghua Zhong Liu Za Zhi, 2003
- Sensitivity to FGF 2  [ **FGF2** ]  disappeared after 7 days although  **FGFR1**  expression was maintained.  Ref: 12746216 Am J Physiol Endocrinol Metab< MedlineTA>Am J Physiol Endocrinol Metab, 2003
- METHODS Immunohistochemistry and confocal microscopy were used to localise FGF 2  [ **FGF2** ]  and  **FGFR1**  in cryosections of the rat retina, both normal retina and retina stressed by exposure to bright continuous light 1000 lux, 24h.  Ref: 12708794 Growth Factors, 2002
- We conclude that TSH stimulates  **FGFR1**  but not FGF 2  [ **FGF2** ]  expression.  Ref: 12746216 Am J Physiol Endocrinol Metab< MedlineTA>Am J Physiol Endocrinol Metab, 2002
- Northern and Western blot analysis and immunohistochemistry were used to screen for the fibroblast growth factor receptor 1  [ **FGFR1** ]  FGF R1 and its high affinity ligand, basic fibroblast growth factor  [ **FGF2** ]  bFGF  [ **FGF2** ] , which could have a role in this recovery process.  Ref: 12626345 Am J Respir Crit Care Med, 2003
- There was also a significant difference in VEGF, bFGF  [ **FGF2** ] , FLK 1, FLG  [ **FGFR1** ]  and MVC between the stage 0 approximately II and stage III approximately IV P <.  Ref: 12795839 Zhonghua Zhong Liu Za Zhi, 2003
- These cells predominantly expressed fibroblast growth factor receptor type 1 FGFR 1  [ **FGFR1** ] , and a minority exhibited basic fibroblast growth factor  [ **FGF2** ]  bFGF  [ **FGF2** ] .  Ref: 12514221 J Neurosci, 2003
- noggin expression is suppressed by  **FGF2**  and syndromic fgfr  [ **FGFR1** ]  signalling.  Ref: 12687003 Nature, 2003
- Colocalisation of  **FGFR1**  and FGF 2  [ **FGF2** ]  could be demonstrated in stressed retina, in the cytoplasm and the axon terminals of photoreceptors.  Ref: 12708794 Growth Factors, 2002
- To detect more dynamic, functional components of the epithelial mesenchymal trophic unit, we evaluated the distribution of perlecan, fibroblast growth factor 2  [ **FGF2** ]  FGF 2  [ **FGF2** ] , and fibroblast growth factor receptor 1  [ **FGFR1** ]  FGFR 1  [ **FGFR1** ] .  Ref: 12388330 Am J Physiol Lung Cell Mol Physiol, 2002
- To understand the significance of this, effects of  **FGFR1**  activation on normal human thyrocyte growth and function in vitro and the regulation of FGF 2  [ **FGF2** ]  and  **FGFR1**  expression have been examined.  Ref: 12746216 Am J Physiol Endocrinol Metab< MedlineTA>Am J Physiol Endocrinol Metab, 2002
- AIM To identify sites of FGF 2  [ **FGF2** ]  action on photoreceptors of the rat retina, by localizing  **FGFR1**  in the intact retina, and to assess the colocalisation of FGF 2  [ **FGF2** ]  with  **FGFR1** .  Ref: 12708794 Growth Factors, 2002
- Immunohistochemical assays for bFGF  [ **FGF2** ] , fibroblast growth factor receptor 1  [ **FGFR1** ]   **FGFR1** , and proliferating cell nuclear antigen were performed.  Ref: 12784342 Cancer, 2003
- Subcellular localization of basic fibroblast growth factor  [ **FGF2** ]  and fibroblast growth factor receptor 1  [ **FGFR1** ]  in pituitary adenomas.  Ref: 12455885 Brain Tumor Pathol, 2002
- As a result of activation of the FGFR  [ **FGFR1** ]  in autocrine cells, changes in cell morphology and an increase in the invasive and tumorigenic properties were observed, however no in vitro or in vivo differential functions between FGF 1 and FGF 2  [ **FGF2** ]  could be identified in this system.  Ref: 12444548 Oncogene, 2002
- **FGF2**  stimulated microvessel formation was inhibited by sequestration of  **FGF2**  via addition of soluble FGF receptor FGFR  [ **FGFR1** ]  chimera protein or anti  **FGF2**  antibodies.  Ref: 12388106 Am J Physiol Cell Physiol, 2003
- Heparan sulfate glycosaminoglycans derived from endothelial cells and smooth muscle cells differentially modulate Fibroblast Growth Factor 2  [ **FGF2** ]  biological activity through Fibroblast Growth Factor Receptor 1  [ **FGFR1** ] .  Ref: 12659634 Biochem J< MedlineTA>Biochem J, 2003
